# Imprinting of Microorganisms for Biosensor Applications

**DOI:** 10.3390/s17040708

**Published:** 2017-03-29

**Authors:** Neslihan Idil, Bo Mattiasson

**Affiliations:** 1 Department of Biology, Faculty of Sciences, Hacettepe University, 06800 Ankara, Turkey; 2 Department of Biotechnology, Lund University, 22362 Lund, Sweden; bo.mattiasson@biotek.lu.se; 3 CapSenze Biosystems AB, 22363 Lund, Sweden

**Keywords:** microorganism imprinting, biosensor, applications

## Abstract

There is a growing need for selective recognition of microorganisms in complex samples due to the rapidly emerging importance of detecting them in various matrices. Most of the conventional methods used to identify microorganisms are time-consuming, laborious and expensive. In recent years, many efforts have been put forth to develop alternative methods for the detection of microorganisms. These methods include use of various components such as silica nanoparticles, microfluidics, liquid crystals, carbon nanotubes which could be integrated with sensor technology in order to detect microorganisms. In many of these publications antibodies were used as recognition elements by means of specific interactions between the target cell and the binding site of the antibody for the purpose of cell recognition and detection. Even though natural antibodies have high selectivity and sensitivity, they have limited stability and tend to denature in conditions outside the physiological range. Among different approaches, biomimetic materials having superior properties have been used in creating artificial systems. Molecular imprinting is a well suited technique serving the purpose to develop highly selective sensing devices. Molecularly imprinted polymers defined as artificial recognition elements are of growing interest for applications in several sectors of life science involving the investigations on detecting molecules of specific interest. These polymers have attractive properties such as high bio-recognition capability, mechanical and chemical stability, easy preparation and low cost which make them superior over natural recognition reagents. This review summarizes the recent advances in the detection and quantification of microorganisms by emphasizing the molecular imprinting technology and its applications in the development of sensor strategies.

## 1. Quantifying Microbial Cells Based on Surface Properties

Antibodies have been used for the identification and quantification of microorganisms. This has been based on affinity binding to cell surface structures. As indicating mechanisms, one has been able to use microbial metabolic activity of the captured cells, or one has used labelled reagents. A problem has been that different microbial strains of a certain species may have different surface structures which leads to that not all cells will react equally to the antibodies used.

When using molecularly imprinted polymers (MIPs) for capturing microorganisms, the same options as discussed for antibodies are available, but besides those, there are other options and challenges as well. By utilizing imprinted polymers with cavities of imprinted cells of the same species/strains as shall be assayed, then size and shape are other features that are utilized in the identification of the target microorganism.

When using MIPs for quantifying microorganisms, some features that are new as compared to when protein molecules are quantified. The cell surface is huge in relation to that of a protein and many different structures might be present on the cell surface. Furthermore, a large surface fitting in a complementary imprint will be exposed to a large number of weak interactions, and maybe some stronger. However, a large number of weak interaction simultaneously will result in very strong interaction. If free cell surface antigens are available in the sample to be analysed one can expect binding of these free compounds, but if/when cells are bound protein molecules that have been captured will be displaced.

In the process of designing the MIP, a range of different monomers can be used. The strategy is normally to equilibrate the imprinting structure with monomers prior to polymerization to start. This is done in order to position monomers with specificity for certain structures so that their positions are frozen after polymerization, thereby improving the probability that the steric arrangement will be suitable for affinity capturing to take place.

The multitude of weak interactions makes it possible that other particulate matters which can establish a large number of interactions to bind even if those are not identical with the print cell. This fact highlights the need for more selectivity in the interactions. The fact that multipoint of attachment makes the binding strong also leads to problems when releasing bound material before the imprinted matter can be reused in a new assay. Many of these features discussed above will be illustrated in this review paper.

## 2. Use of Molecularly Imprinted Polymers in Affinity Recognition of Microbial Cells

The detection and identification of pathogenic bacterial strains present in soil, marine and estuarine waters, the intestinal tract of animals, or water contaminated with fecal matter is of great importance in many fields such as medicine, water and food safety and security [1,2]. Among various known bacterial strains, *Staphylococcus aureus* is the cause of serious infections and remains a critical threat to public health [3]. Especially, methicillin-resistant *Staphylococcus aureus* (MRSA), resistant to methicillin and other β-lactam antibiotics, have been the cause of difficult-to-treat infections in humans [4]. Another important organism in this context is *Escherichia coli* (O157:H7) which is a predominant enteropathogen and has a very low human infectious dose, since as few as ten cells are required to cause an infection [5]. It is well documented that *Listeria monocytogenes* has long been recognized as a cause of food poisoning which can lead to very serious diseases or even death. On the other hand, the identification and detection of pathogenic bacterial strains have become increasingly important in clinical diagnosis and treatment as well as prevention the spreading of responsible strains [2]. An emerging field is those bacteria with potential use in bioterrorism such as *Bacillus* *anthracis.*

Infectious diseases, especially those caused by life-threatening pathogenic bacterial strains, have become a great concern due to these organisms cause death and increased morbidity in hospitalized patients. Even though several attempts have been made to develop vaccines and antimicrobial agents, these infections still remain as a major challenge because of the existence of new and multi drug resistant microorganisms [2]. On the other hand, current techniques used for detecting pathogenic bacterial strains are based on plating/culturing, biochemical tests, serological and immunological assays and genotypic analysis [2,5,6]. These techniques have some disadvantages such as being time-consuming, laborious and expensive, lacking sensitivity and specificity in comparison with selective, sensitive, multi-analyte testing capacity, speed and cost-effectiveness features of new analytical methods [2,6]. Therefore, the point is that rapid, portable and reliable analytical detection is of primary importance to be applied for early and accurate diagnosis.

Biosensor technology has been introduced to be promising tools for microbial detection in complex media (blood, serum, urine, food, water, etc.) with minimum sample pre-treatment. This technology is an approach including the ability to provide specific quantifiable data related with a broad spectrum of analytes [1]. It also is suitable for high-throughput monitoring, label-free detection, short response time [7], real-time analysis, simple sample preparation, low detection limits [6]. Biosensors are comprised of a biological recognition element in close conjunction with a transducer that transforms biological response resulting from the interaction of the analyte with the bioreceptor into measurable signal [2]. Biosensor-based techniques improves their detection performance by means of considered properties, thereby holding great promise for a variety of applications in the fields of clinical diagnostics, food and water analysis, product quality and process control in the agricultural and food industry, agricultural and industrial processing, bioprocess and environmental monitoring [1,6,7].

Many efforts to create innovative methods to be used for microbial detection includes use of various materials such as silica nanoparticles [8], microfluidics [9], liquid crystals [10], carbon nanotubes [11] which could be integrated to sensor technology. Receptors, aptamers, enzymes, nucleic acids, antibodies and lectins are placed among biological recognition elements in this technology [12,13]. In many of the studies dealing with microbial detection, antibodies were used in order to recognize and separate target cells. Antibodies, serving as specific affinity ligands, contribute to generate highly selective and sensitive bio-sensing systems. However, natural receptors tend to denature in conditions outside the physiological range such as elevated/lowered temperature, strongly acidic/basic conditions, presence of organic-rich solvents etc. [3,12]. One additional limitation of biological recognition structures is the fact that they are easily degradable by e.g., proteases. This limits their use in non-sterile media. They have also additional disadvantages by the fact that usage of animals is essential for the production and there is shelf-life restrictions and special storage requirements. Moreover, handling of natural receptors is not as cost-effective as handling synthetic receptors due to these limitations [3]. The use of artificial receptors as well as natural ones coupled to biosensor technology allows the possibility of creating highly selective and sensitive sensing systems with low cost. Therefore, this alternative approach has recently attracted the interest of researchers and it has had a profound impact on the development of biosensors for various applications. With this approach, it is possible to generate well-established artificial affinity ligands and develop effective biosensing methodologies for the detection of microorganisms. In literature, it is highlighted that molecular imprinting technology is presently a wide-spread effort to fill the major gap in different fields and offers artificial recognition systems exhibiting superiority over natural ones. This technology also provides tools to form specific recognition cavities in synthetic polymer matrix which are complementary in size, shape and/or chemical functionality to the template [3]. MIPs are comprised of a tailor-made polymer matrix obtained by polymerization of functional monomers with cross-linkers in the presence of a template molecule. In addition to these, this pre-polymerization solution contains initiator in an appropriate solvent. In order to initiate polymerization, UV irradiation or temperature treatment is necessary. After the entire polymerization process is completed, the stable matrix is washed for the removal of template and unreacted monomers, by this way creating selective recognition cavities which are then able to rebind the target molecule of interest selectively and reversibly. These cavities are designed by using most often the target molecule itself or very close structural analogues of it and therefore imprinted polymer having a memory for the target molecule is produced [3]. The correct placement of the functional groups and the size-shape characteristics of binding site have considerable effect on rebinding of the target molecule. After the removal of the template, the shape stability of the polymer network could be attributed to high degree of cross-linking and the functional groups arranged in proper positions which makes it possible to rebind the template.

The corresponding sites located in imprinted polymer have been generated by: (i) covalent; (ii) non-covalent and (iii) semi-covalent interactions [14]. Binding of the template into the polymer or also removal of this molecule from the polymer matrix and rebinding of this molecule depend on same chemical interactions [15]. There are some drawbacks in covalent imprinting such as requirement of cleaving the covalent bond and a limited number interactions play a key role when the interactions between the template and polymeric structure were taken into consideration. In this imprinting method, strong covalent binding is formed which results in obtaining binding regions. However, it is not useful in most cases because of the obstacles in rebinding of the target molecules. Non-covalent imprinting is suggested to overcome these shortcomings. This approach involves interactions such as, hydrogen bonding, van der Waals forces, dipole-dipole and hydrophobic interactions. This method also contributes to produce template-free recognition sites, and thus it is the most frequently preferred due to its appropriateness on extracting the template from the rigid polymer and making it possible to rebind the target molecule with the same approach. Therefore, it has become feasible for a great number of target molecules and found wide-spread use in various applications. The concept of semi-covalent imprinting relies on the combination of covalent and non-covalent imprinting [16]. Covalent binding is formed when the template molecule is trapped during the synthesis of the polymer. On the other hand, following the removal of the template, the target molecule can bind to its complementary cavity in a reversible manner via non-covalent binding. As mentioned above, non-covalent binding approach seems rather simple and more effective in the preparation of MIP when compared to the other methods.

Molecular imprinting technology has potential applications in many fields such as catalysis, separation, purification, detection and drug delivery for a wide range of target molecules from small to macromolecules and particulate matter including microorganisms [17,18,19]. A number of reports focused on the synthesis of polymers via imprinting applied for aminoacids [20], peptides [21], carbohydrates [22], nucleotides [23], hormones [24], drugs [25], toxins [26], pesticides [27].

## 3. Imprinting of Microorganisms

In the recent literature, there are many publications describing successful imprinting of small molecules. However, from the standpoint of much larger and complex structures or molecules [28], especially imprinting of whole cells is an ongoing challenge due to some characteristics and natural structures of microorganisms [3,19]. The main complexity in imprinting of microorganisms in comparison to less complicated organic substances is the huge size resulting in some difficulties while removing the bound cells from template-shaped cavities. Furthermore, imprinted cavities have a significant effect on the recognition of microorganisms since transport of the cells into the matrix is performed slowly and resultant diffusion problem extends the response time of the system. It is important to optimize the MIP polymer composition by varying the monomers and their ratios used in polymerization and/or the method applied for designing the polymer matrix in order to overcome these limitations. For this purpose, MIPs were synthesized by increasing the degree of cross-linking and generating well-patterned surfaces which facilitate the transport of the microorganisms to and from the corresponding sites. The surfaces of microbial cells have various structures which exhibit several differences in terms of morphology and functionality. These characteristics of the surfaces present in every microorganism show extensive diversity in each species. Accordingly, a crucial prerequisite is the choice of proper functionality in the sites. Following this, the properties of functional monomers have to be taken into consideration because these monomers interact with the groups of cell surfaces having complementary functionalities. It is also necessary to manage to create functionally adapted cavities by maintaining size and shape characteristics of microorganisms in the polymer matrix. Another requirement is to arrange the right functionality in established sites [29]. Taking the imprinting of microorganisms into account, reversible and selective re-inclusion is more significant. When the imprinted cavities are occupied by microorganisms, removal of bound material becomes troublesome because of steric hindrance and therefore the property of reversible binding will be lost. It is comparatively hard to remove the template from imprinted cavities by maintaining their conformation on the polymer surface in the absence of template. A further fact that has to be kept in mind is that microbial cells have, in relation to individual protein molecules, a large number of interactions with the MIP. This leads to that also weak interactions become important due to the effects of multipoint of attachment. Therefore, new criteria to create high selectivity need to be developed.

A wide variety of functional monomers such as those forming pyrrole conducting polymers [30,31,32], polyurethane layers [33,34], self-assembled monolayers [35,36] and sol-gels [37,38] have been used in the design of imprints against microorganisms [12]. The imprinting of whole microorganism were successfully achieved by creating selective recognition cavities [12,39] both on polymeric beads [40,41,42] and in polymeric films via stamping method [34,43].

Vulfson and co-workers made an attempt dealing with bacteria-mediated lithography and published the first successful live cell imprint report. In their study, *Listeria monocytogenes* and *Staphylococcus* *aureus* were selected as templates. The bacterial cells attaching on the surfaces were formed due to the tendency of microorganisms to accumulate at the interface between the aqueous and organic phases. After removing the microorganism, surface imprints were patterned in size and morphology. It has to be underlined that the proposed technique addresses merely the features of size and morphology without surface chemistry. In addition, another missing side of this pioneering research is to not include rebinding studies in order to indicate imprinting selectivity [40].

In bulk imprinting, the template structure (microorganism) is added to the pre-polymerization mixture along with monomer, crosslinker, initiator and solvent. Following polymerization and template removal, it leaves behind homogenously distributed cavities in the whole polymer matrix. This approach is more appropriate for imprinting of relatively small molecules [44]. The dimensions of microorganisms limit the transport of microorganisms into the polymer matrix. Cohen et al. made an attempt in order to detect microorganisms in water. In that study, imprinting of whole cells of microorganisms with different morphologies and outer surface chemistry was performed using thin sol-gel films of organically modified silica (ORMOSILS). These imprinted films having complementary cavities did recognize the target bacterial strains, and thus high adsorption affinity was obtained along with high selectivity [38].

Another strategy is surface imprinting in which thin polymeric films were formed along with the production of template-imprinted cavities on the surface of the polymer. The template stamp is prepared, and then pressed onto the polymerizing surface. The stamping is performed on the transducer and therefore provides the formation of more robust devices easily employable for measurements. This approach is particularly successfully applied for imprinting of large-size biomolecules as well as microorganisms (i.e., yeast, bacteria). Among known imprinting techniques, surface imprinting provides a feasible route to manage successful results in the detection of microorganisms because of enabling the transport of cells to and from the cavities [3,12].

Surface imprinted polymers could be prepared by a self-assembly process. In a previous study, *E. coli* was imprinted on polymeric microspheres. The aqueous suspensions of bacterial cells were added to water containing *N*-acrylchitosan. This solution was mixed with an oil phase for the production of microspheres. Following cross-linking the chitosan matrix, the bacterial strain was extracted and analysis was performed by fluorescence microscopy and verified that the microbeads have the ability to differentiate rod-shaped *E. coli* and spherical *Micrococcus luteus* [35].

A number of publications focused on revealing alterations to the surface imprinting concept by making extra contributions to the self-assembly process. Different from previously mentioned imprinting materials, overoxidized polypyrrole (PPy) films are used in order to detect anionic species in electrochemical sensing platforms. The stable electrical conductivity property of conducting polymers makes doping of the anionic bacterial strains easy during elecropolymerization process. Tokonami et al. (2014) developed highly selective devices for the detection of both Gram-negative (*E. coli* and *Pseudomonas aeruginosa*) and Gram-positive bacteria (*Bacillus subtilis* and *S. aureus*) exploiting their surface chemistry [31].

Another strategy is molding which includes the immobilization of the template molecules on a solid substrate. For example, Qi et al. described a similar strategy in order to prepare bacteria-imprinted polymers by this technique. In this study, indium tin oxide electrodes were covered by a nanocomposite film of chitosan and reduced graphene oxide sheets by electrodeposition. Sulfate-reducing bacteria present in marine environments were selected as template microorganism and attached on this film by physical adsorption [19].

The first study to implement surface-imprinted polymers in microbial fuel cells was carried out for recognition of green algae (*Chlamydomonas reinhardtii*). The algae cells were immobilized on platinum foils coated by an algae-imprinted polymeric thin films of ethylene-covinyl alcohol (EVAL) by molding method. Sputter-coated platinum electrode was selected since the surface of the electrode could be well-patterned by sputtering on a planar plastic thin film for the production of microcontact imprinting. Fluorescence spectrometry was performed for the determination of recognition capacity of the algae-imprinted cavities [45]. Following this study, further investigations were performed to study the effects of polymer concentration on microcontact imprinting of the same microorganism. For this purpose, various concentrations of EVAL were tested due to their effect on both morphology and efficiency of imprinted films [46]. In another study, it was indicated that algal metabolism may affect the generation of electricity in biofuel cells. For this purpose, *C. reinhardtii* was microcontact imprinted onto EVAL films. The production of hydrogen catalyzed by hydrogenase of the microorganism was measured electrochemically [47].

Microcontact imprinting, an alternative approach to design well-defined surfaces, is based on the polymerization taking place between a target stamp and the surface onto which the MIP shall be formed. This method generated much attention due to offering some crucial advantages over the other molecular imprinting methods. One unique feature is that the print structure is never fully embedded in the polymer, thereby making it easier to remove the template and to rebind macromolecular and even particulate structures [48].

In a study, the effects of adsorption to bacteria imprinted polymer matrices on gene expression were studied. The purple bacteria *Rhodobacter sphaeroides* was selected as a template and it was immobilized on a glass slide for the production of the bacterial stamp and then microcontact-imprinted onto EVAL. The surfaces of the *R*. *sphaeroides*-imprinted (RsIPs) and non-imprinted (NIPs) EVAL thin films were analyzed by Raman spectrometry and scanning electron microscopy. The expression of the nitrogenase playing a key role in nitrogen fixation gene of *R. sphaeroides* attached on EVAL thin films was also measured by the quantitative reverse transcription polymerase chain reaction (qRT-PCR) for the confirmation [49].

## 4. Molecular Imprinting of Microorganisms in Biosensor Platforms

MIPs have been considered as effective alternatives to natural recognition systems in order to improve detection of microorganisms with enhanced selectivity. Molecular imprinting (MIP) technology is a powerful tool in the development of selectively working biosensor systems having polymeric MIP structure [50]. In order to create applicable sensor surfaces, the coating material has to be resistant against organic solvents and applicable for a wide range of target molecules, recognize template molecules sensitively and show high stability. MIPs open up the possibility to meet the requirements on improving adapted sensor surfaces and provide the improvement of the detection efficiency. Microorganisms with unique structures and conformations can be discriminated using molecularly imprinted materials in biosensing systems in/on which sterically and functionally complementary sites are present. In order to develop sensing tools exhibiting high operational performance, the optimization of components have to be done to generate reversible interactions between binding cavities and microorganisms.

It is not easily possible to construct well-patterned cavities during an imprinting process due to the non-rigid structure of microorganisms. Even though in principle possible, the imprinting of microorganisms are still challenging when performed by traditional MIP methodologies. In this concept, highly cross-linked polymers were formed and this makes the placement of bacterial cells complicated and restricts the access of them to the interaction regions embedded in the interior of imprinted polymers. In order to overcome intrinsic limitations, efforts to improve molecular imprinting technology were directed towards surface imprinting as a promising approach. This approach has the advantage of integration of MIPs to the sensor platforms and thus surface-molecularly imprinted sensors were designed. The polymers synthesized by this technique covered the sensor as thin MIP-films in order to fabricate selective sensing tools for different template molecules [51,52,53].

Biosensor technology provides the opportunity of easy miniaturisation and flexible operation for the improvement of accomplished biosensors [54]. On the other hand, several transducer devices have been proven to be suitable for on-line monitoring used in order to detect and quantify microorganisms [36,55,56,57]. In recent years, many attempts have been made to detect whole bacterial cells especially by using impedimetric and optical sensors [2].

Surface-plasmon resonance (SPR)-based optical biosensors are able to measure the changes in refractive index near the surface of a chip related with the interaction between the analyte and biorecognition element.

Electrochemical sensors are capable of measuring electrochemical changes which take place with the interaction between chemicals and a sensing surface of the detecting electrode. The electrical changes can be based on a change in the measured voltage between the electrodes (potentiometric transducer), a change in the measured current at a given applied voltage (amperometric transducer), or a change in the ability of the sensing material to detect changes in the electric field (Impedance including capacitance/conductance). Among electrochemical biosensors, capacitive biosensors have attractive properties such as label-free, real-time and rapid detection of macromolecules along with both high selectivity and low detection limit [58]. The capacitance measurement principle of these sensors relies on the changes in dielectric properties of the electrode surface. The interaction between analyte of interest and electrode surface leads to a decrease in capacitance [59,60].

## 5. Applications

Dickert and co-workers [34] were the pioneers of attempts at whole cell imprinting integrated in biosensor platforms. In their first report, a model yeast cell *Saccharomyces cerevisiae* was imprinted in polyurethane and honeycomb-like yeast imprints were formed by stamping and the measurements were performed on mass sensitive sensor (quartz microbalance—QCM—devices). The proposed sensor was prepared via sol-gel method including imprinted films composed of polyurethane and alkoxide imprinted with whole yeast cells. It was possible to detect and quantify yeast cells in cell concentrations between 10^4^ and 10^6^ CFU/mL at flow rates of 10 mL/min. In comparison to polyurethane, it was determined that alkoxide sol-gel layers were more robust on which low sensor responses were obtained using the template microorganism. In the second report, surface-imprinted polyurethanes were generated in order to detect *E. coli* using QCM [61].

Acrylic acid and acrylamide derivative functional monomers could be used for the imprinting of microorganisms [34]. However, when these functional monomers are used for the imprinting, exposure to UV radiation or heat treatment is necessary for initiating of the polymerization process. Other than these, the whole cells of microorganisms were imprinted on thin films of organically modified silica prepared using sol-gel technique [38]. These alternative bacteria-imprinted films produced by sol-gel method includes polycondensation of silanes and uses mild conditions in comparison to the acrylate polymerization. Crosslinked and robust MIP films obtained through sol-gel techniques are resistant to harsh conditions such as thermal and solvent stresses. These matrices show high affinity against microorganisms in terms of both morphology of the imprinted cavity and functional groups located on the surface of the microorganism. Extraction of the template results in sterically and functionally adapted binding cavities in or on a porous matrix. Resultant MIP films were capable of differentiate between bacterial species with the same geometrical form [56].

Cohen et al. used thin films of organically modified silica (ORMOSILS) formed by sol-gel technique in order to imprint whole cells of different bacterial strains. These materials have gained great interest for the creation of sensing devices with the combination of molecular imprinting [62]. They provide a platform to generate simple, versatile and long term stable biosensors exhibiting good selectivity with high sensitivity [63]. *Deinococcus radiodurans*, *E. coli*, *B. subtilis* and *Sphaerotilus natans* were selected as templates for imprinting since they have different morphologies and show size variations. The developed materials indicated high adsorption affinity towards the target microorganisms and were promising tools for rapid bacterial detection from water [38].

Apart from functional monomers such as acrylic acid and sol-gel based ones, monomers used to form conducting polymers have found wide application in order to imprint biomolecules including microorganisms [31,32,64]. The properties of easy preparation and high conductivity makes these polymers appropriate for designing biosensors. Furthermore, their desirable feature is associating anionic molecules in the structures to compensate for the cationic charges introduced by the polymer backbone. This contribution makes these biocompatible polymers being applicable for the immobilization of biomolecules (enzymes, DNA etc.) [64]. Among conducting polymers, polypyrrole (PPy) based ones are extensively implemented to the imprinting studies. These polymers also used for developing bacterial templates on the surface of an overoxidized polypyrrole film using both gram-negative and gram-positive bacteria [31]. The functional groups present on the outer surface of the bacterial cell wall such as carboxyl, phosphate and hydroxyl groups correspond to the anionic sites. By this way, docking of the bacteria into a positively charged conducting polymer easily occurs due to the presence of related functional groups of the microorganism [64]. When the entire process of microorganism imprinting is taken into account, it is obvious that capture of the target cells operates according to expectations. The binding may, however, be too firm since there are problems in releasing bound material. This is a serious limitation since the concept of biosensors is to reuse the sensor surface for repeated analyses. If it shall be used as a disposable entity, then it becomes more like a dipstick. The missing removal is one of the most urgent needs to be resolved issue. The use of conductive polymers in microorganism imprinting offers a solution to this existing problem. The template was taken out from the polymer matrix with electrochemical overoxidation protecting the shape formed on the polymer matrix [31,56].

In a previous study, *P. aeruginosa* was selected as target microorganism and was transferred on the surface of overoxidized polypyrrole (OPPy) film. Then, it was determined that proposed approach provided highly selective and rapid detection of the target bacterial strain by application of QCM even in suspensions of bacterial mixtures including *Acinetobacter calcoaceticus*, *E. coli*, and *Serratia marcescens*. The successful detection could be performed at concentrations as low as 10^3^ CFU/mL within 3 min. The real sample experiments were carried out with apple juice to verify whether the prepared system works and the measurable concentration range in apple juice was pointed out between 10^7^ to 10^9^ CFU/mL for tested bacterial strain [30].

In another study, *B. subtilis* endospores were imprinted using conducting polymer films. The resultant imprinted films were prepared by holding endospores on the surface of glassy carbon electrodes. A polypyrrole layer presented on the top of this electrode followed by poly(3-methyl-thiophene) layer. Then, the films were sequentially deposited by electropolymerization. Measurements of the changes in the electrochemistry of the conducting polymer film depending on endospore binding were carried out in order to detect the endospores [65].

Electropolymerization offering some advantages compared to more common preparation procedures of MIP films could be effectively integrated to the biosensor platforms [53]. Qi et al. created an impedimetric biosensor with bacteria-mediated bio-imprinted films in order to develop rapid detection devices for sulfate reducing bacteria. Chitosan (CS) was preferred due to its unique features such as biodegradability, low toxicity and good biocompatibility which makes them appealing alternatives in designing biosensors. A nanocomposite film with reduced graphene sheets (RGSs)/CS was electrodeposited on the surface of an indium tin oxide (ITO) electrode, and the RGSs/CS hybrid film behaved as a recognition element for the binding of target microorganism. The role of RGSs was increasing the conductivity of the film. Following bacterial attachment on the RGSs-CS nanocomposite films, a layer of CS was electrodeposited around the immobilized microorganism. After template removal, binding studies were performed by monitoring the impedance change of the biosensor with a detection limit of 0.7 × 10^4^ CFU/mL. The selectivity studies carried out using bacteria-mediated bioimprinted film with *M. luteus*, *S. aureus*, *Vibrio anguillarum* and *Vibrio alginolyticus* confirmed the selectivity of the developed sensor [19].

Roy et al. developed an electrochemical sensor with Ag-ZnO bimetallic nanoparticle and graphene oxide nanocomposite using stamping method. The purpose of this study was to detect, remove and kill *E. coli* cells. The nanocomposite was selected as a platform in order to imprint target microorganism. The MIP-modified sensor was capable of detecting *E. coli* with limit of detection 5.9 CFU/mL, removing 98% of cells. In addition, 16 cm^2^ modified area of the plate is sufficient to kill bacteria in a concentration of 10^5^ CFU/mL. The real-sample experiments were performed with waste water samples. It was demonstrated that *E. coli* cells could be detected in the complicated matrix of waste water with the recovery of 92%–98%. The generated sensor offered simple, low-cost, short time analysis [5].

**Figure 1:**
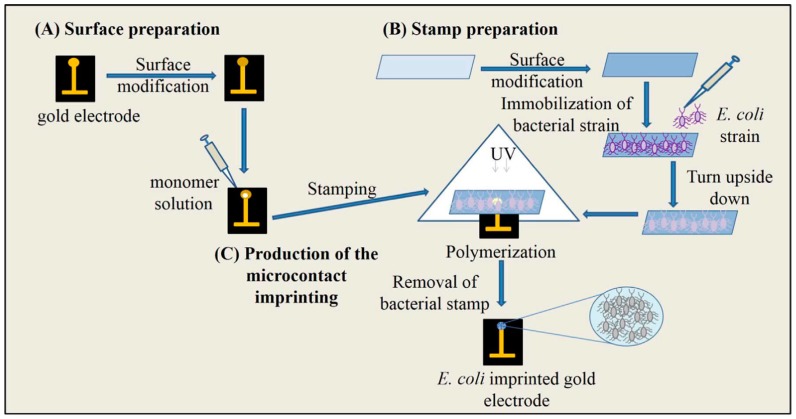
Schematic representation of microcontact imprinting of *E. coli* onto the polymer modified surfaces. (A) preparation of electrode surface; (B) preparation of bacteria stamps; (C) production of the microcontact imprinting (reproduced from [60] with permission).

In our previous study, a label-free, selective and sensitive microcontact imprinted capacitive biosensor was generated in order to detect *E. coli*. The recognition of the target cells was successfully achieved by this sensor prepared with the combination of microcontact imprinting method and capacitive biosensor technology. After preparation of bacterial stamps, microcontact-*E. coli* imprinted gold electrodes were produced using an amino acid based recognition element, *N*-methacryloyl-l-histidine methylester (MAH) and 2-hydroxyethyl methacrylate (HEMA) as monomers and ethyleneglycol dimethacrylate (EGDMA) as crosslinker under UV-polymerization conditions. *E. coli*-imprinted capacitive electrodes were prepared using microcontact imprinting by bringing the modified surface with the vinyl groups of the electrode and the monomer solution in contact with the *E. coli*-immobilized on the glass slide like a sandwich (Figure 1). 

**Figure 2:**
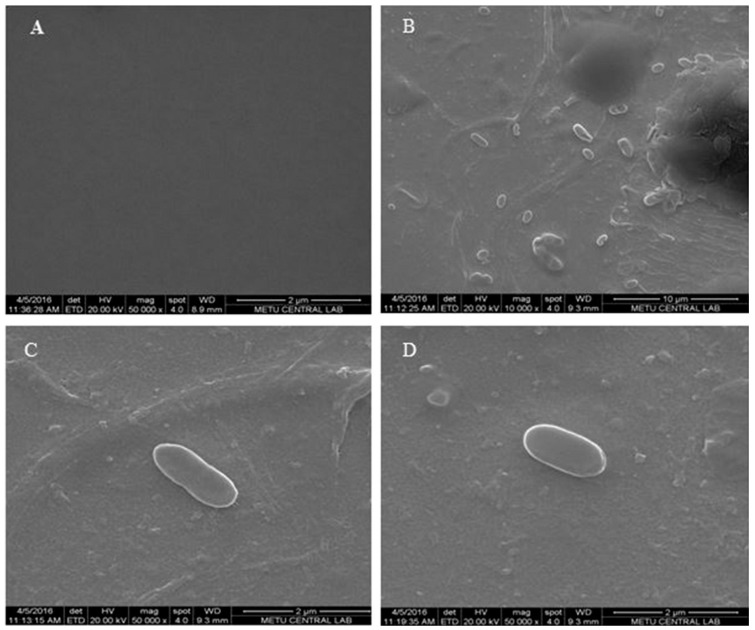
SEM images of bare gold electrode (**A**) −50,000×, and *E. coli* imprinted electrode (**B**) −10,000×, (**C**,**D**) −50,000× magnifications (reproduced from [60] with permission).

**Figure 3:**
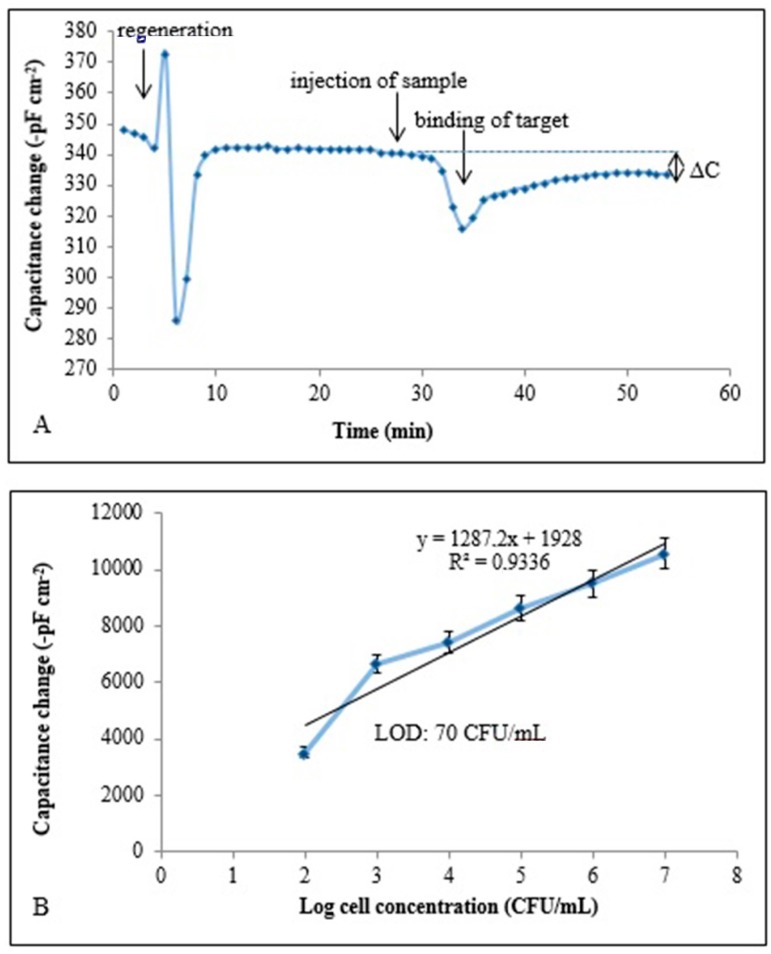
(**A**) Capacitive sensorgram of the *E. coli* imprinted electrode (concentration of *E. coli*: 1.0 × 10^7^ CFU/mL), ΔC: change in capacitance; (**B**) calibration curve of *E. coli* obtained at a concentration range of (1.0 × 10^2^–1.0 × 10^7^ CFU/mL) under optimum conditions, LOD: limit of detection, flow rate: 100 μL/min; sample volume: 500 μL; running buffer: 10 mM sodium phosphate, pH: 7.4; regeneration buffer: pure ethyl alcohol, 10 mg/mL lysozyme solution (in 10 mM Tris-HCl buffer, pH 8.0, with 1 mM EDTA) and 50 mM glycine-HCl, pH 2.5; T 25 °C. (Reproduced from [60] with permission).

SEM images in Figure 2 also show the changes in the morphology of the polymeric surface on the *E. coli*-MIP electrode in different magnifications, Figure 2B–D also show the captured cells. The unique combination of these two techniques provides selective detection with a detection limit of 70 CFU/mL. Capacitive sensorgram of the *E. coli* imprinted electrode and calibration curve of *E. coli* obtained at a concentration range of (1.0 × 10^2^–1.0 × 10^7^ CFU/mL) were given in Figure 3A,B, respectively. The recognition of *E. coli* took place by the functional group of MAH in the imprinted film and shape-complementary cavities to *E. coli.* The generated capacitive sensor has high selectivity and was able to distinguish *E. coli* when present together with competing bacterial strains which are known to have similar shape. In addition, the prepared sensor has the ability to detect *E. coli* with a recovery of 81%–97% in e.g., river water [60].

Yilmaz et al. developed microcontact imprinted SPR and QCM sensor surfaces for the detection of *E. coli*. They used an amino acid-based recognition element, MAH, as functional monomer. Recognition occurred via of functional group (MAH) in imprinted film and shape-complementary cavities belonging to *E. coli* on the imprinted film surface. The schematic representation of microcontact imprinted SPR and QCM sensor surfaces developed in this study. Characterization studies were performed with SEM and AFM. In that study, the limit of detection (LOD) and the limit of quantification (LOQ) of QCM system were found as 3.72 × 10^5^ CFU/mL and 1.24 × 10^6^ CFU/mL, respectively. LOD and LOQ of SPR system were also calculated and were found as 1.54 × 10^6^ CFU/mL and 5.13 × 10^6^ CFU/mL, respectively. Real-time responses and linear regions when plotting the relation of log cell conc. vs. the signal registered of SPR and QCM. The researchers preferred the apple juice spiked with *E. coli* at different concentrations in the range of 0.5–4.0 McFarland (approx. 1.5 × 10^8^ to 12 × 10^8^ CFU/mL) as real sample in order to verify the proposed SPR and QCM sensors for detecting *E. coli* [36].

There are several publications in which bio-sensing systems are based on application of molecular imprinting. In recent years, imprinted polymers have gained increasing attention of researchers in different fields. In these platforms, imprinted materials are used as artificial recognition elements integrated with a transducer component. The real time detection of an interaction occuring between the unique binding sites and the target molecule was a stimulating feature in order to improve such systems as options to conventional methods. Due to some difficulties with proper selective detection of microorganism with cell surface imprinted materials, the development of effective sensing systems still remains to be addressed. The representative examples of microorganism detection using MIP based materials in sensor applications in literature were shown in Table 1.

MIPs show selectively high affinity towards target molecule/cell selected to be used in the imprinting process [66]. These polymers have higher physical robustness, strength, resistance to elevated temperature and inertness against organic solvents in comparison with natural systems [66,67]. Furthermore, their production is less expensive and storage life is higher with preserving their capacity [66].

When the limitations were taken into consideration, formation of new MIPs has complicated challenges which result mainly from the fact that all target molecules/cells are different from each other with the requirement of various functional monomer and cross-linker combinations [68]. The accurate choice and adjustment of the polymer concentration composition provides enough binding regions complementary to the functional groups of the target molecule/cell. The production of favorably developed MIPs is restricted because of inefficient removal of stamp from the polymer surface. In some cases, strong removal methods can be necessary and this sometimes leads to damage binding region of target molecule/cell while fully removal of the template.

## 6. Concluding Remarks

The present review summarizes the status with regard to biosensor-based assays of microorganisms by registration of their binding to artificial affinity sites created via use of molecularly imprinted polymers. Several of the assays are direct binding assays, i.e., one can register the captured cells directly and thereby get an opinion about the number of cells (often as CFU) per mL. The assays are quick and that is certainly a strong feature. A limitation is still the relatively poor knowledge base on how to design the best affinity cavity to selectively capture and quantify particular microbial cells. Thus, the data so far generated give clear indications of possibilities for an interesting future development of this relatively new approach to quantify microorganisms (most often pathogenic) in our environment.

When comparing microorganism analysis to that of small molecules or even proteins, studies on microorganisms lag far behind with regard to development of the particular analytical field. At the same time it is encouraging to follow how the protein assays have developed from a very low level with an accelerated pace into advanced analytical systems. It is therefore a not too brave guess that analytical devices based on MIPs will undergo a similar development as that of protein analysis.

## Figures and Tables

**Table 1:** Representative examples of microorganism detection using MIP based materials in sensor applications.

Template	Functional Monomer	Imprinting Technique	Determination Technique	LOD/LOQ	Ref.
*Saccharomyces cerevisiae*	Polyurethane	Surface imprinting	QCM	10^4^–10^6^ CFU/mL	[34]
*Saccharomyces cerevisiae*	Titanium ethylate	Surface imprinting	QCM	10^4^–10^6^ CFU/mL	[34]
*Escherichia coli*	Polyureathane	Surface Imprinting	QCM	0.1 mg/mL	[61]
*Deinococcus radiodurans*	Tetraehoxysilane	Bulk imprinting	Fluorescence	10^7^–10^9^ CFU/mL	[38]
*Escherichia coli*	Tetraehoxysilane	Bulk imprinting	Fluorescence	10^7^–10^9^ CFU/mL	[38]
*Bacillus subtilis*	Tetraehoxysilane	Bulk imprinting	Fluorescence	10^7^–10^9^ CFU/mL	[38]
*Sphaerotilus natans*	Tetraehoxysilane	Bulk imprinting	Fluorescence	10^7^–10^9^ CFU/mL	[38]
*Pseudomonas* *aeruginosa*	Pyrrole	Electro polymerization	Fluorescence	-	[32]
*Escherichia coli*	Pyrrole	Electro polymerization	QCM	10^3^–10^9^ CFU/mL	[32]
*Pseudomonas* *aeruginosa*	Pyrrole	Electro polymerization	QCM	10^3^–10^9^ CFU/mL	[32]
*A. calcoaceticus*	Pyrrole	Electro polymerization	QCM	10^3^–10^9^ CFU/mL	[32]
*S. marcescens*	Pyrrole	Electro polymerization	QCM	10^3^–10^9^ CFU/mL	[32]
*Escherichia coli*	Tetraehoxysilane	Bulk imprinting	QCM	10^2^ CFU/mL	[37]
Sulphate reducing bacteria	Chitosan reduced graphene	Electro polymerization	EIS	10^4^–10^8^ CFU/mL	[19]
*Bacillus subtilis* endospore	Pyrrole	Electro polymerization	EIS	10^4^–10^7^ CFU/mL	[65]
*Escherichia coli*	Silane	Stamping	Electrochemical sensor	5.9 CFU/mL	[5]
*Escherichia coli*	MAH	Microcontact Imprinting	Capacitive biosensor	70 CFU/mL	[60]
*Escherichia coli*	MAH	Microcontact Imprinting	QCM	1.54 × 10^6^ CFU/mL	[36]
*Escherichia coli*	MAH	Microcontact Imprinting	SPR	3.72 × 10^5^ CFU/mL	[36]

CFU: colony forming unit, EIS: electrochemical impedance spectroscopy, MAH: *N*-methacryloyl-l-histidine methylester, QCM: quartz crystal microbalance, SPR: surface plasmon resonance.
